# Feasibility, usability and acceptability of a novel digital hybrid-system for reporting of routine maternal health information in Southern Tanzania: A mixed-methods study

**DOI:** 10.1371/journal.pgph.0000972

**Published:** 2023-01-12

**Authors:** Regine Unkels, Fatuma Manzi, Ntuli A. Kapologwe, Ulrika Baker, Aziz Ahmad, Rustam Nabiev, Maria Berndtsson, Jitihada Baraka, Claudia Hanson, Atsumi Hirose

**Affiliations:** 1 Department of Global Public Health, Karolinska Institutet, Stockholm, Sweden; 2 Health System, Policy and Economic Evaluations, Ifakara Health Institute, Dar es Salaam, Tanzania; 3 Health, Social Welfare & Nutrition Services, President’s Office - Regional Administration and Local Government, Dodoma, Tanzania; 4 UNICEF Tanzania Country Office, Dar es Salaam, Tanzania; 5 SHIFO Foundation, Stockholm, Sweden; 6 Department of Disease Control, Faculty of Infectious and Tropical Diseases, London School of Hygiene and Tropical Medicine, London, United Kingdom; 7 School of Public Health, Imperial College, London, United Kingdom; Bangladesh Institute of Development Studies, BANGLADESH

## Abstract

Health information systems are important for health planning and progress monitoring. Still, data from health facilities are often of limited quality in Low-and-Middle-Income Countries. Quality deficits are partially rooted in the fact that paper-based documentation is still the norm at facility level, leading to mistakes in summarizing and manual copying. Digitization of data at facility level would allow automatization of these procedural steps. Here we aimed to evaluate the feasibility, usability and acceptability of a scanning innovation called *Smart Paper Technology* for digital data processing. We used a mixed-methods design to understand users’ engagement with *Smart Paper Technology* and identify potential positive and negative effects of this innovation in three health facilities in Southern Tanzania. Eight focus group discussions and 11 in-depth interviews with users were conducted. We quantified time used by health care providers for documentation and patient care using time-motion methods. Thematic analysis was used to analyze qualitative data. Descriptive statistics and multivariable linear models were generated to compare the difference before and after introduction and adjust for confounders. Health care providers and health care managers appreciated the forms’ simple design features and perceived *Smart Paper Technology* as time-saving and easy to use. The time-motion study with 273.3 and 224.0 hours of observations before and after introduction of *Smart Paper Technology*, respectively, confirmed that working time spent on documentation did not increase (27.0% at baseline and 26.4% post-introduction; adjusted p = 0.763). Time spent on patient care was not negatively impacted (26.9% at baseline and 37.1% at post-intervention; adjusted p = 0.001). Health care providers described positive effects on their accountability for data and service provision relating to the fact that individually signed forms were filled. Health care providers perceived *Smart Paper Technology* as feasible, easy to integrate and acceptable in their setting, particularly as it did not add time to documentation.

## Introduction

Quality and timely health information is crucial to strengthen health systems [1, 2] and to monitor achievements of nationally and internationally agreed targets such as the *Sustainable Development Goals* [3]. Although population-based surveys are the mainstay to generate health data [4], *Health Management Information System* (HMIS) data is increasingly valued as it is continuously available and less cost-intensive than survey data [2, 4].

**Fig 1:**
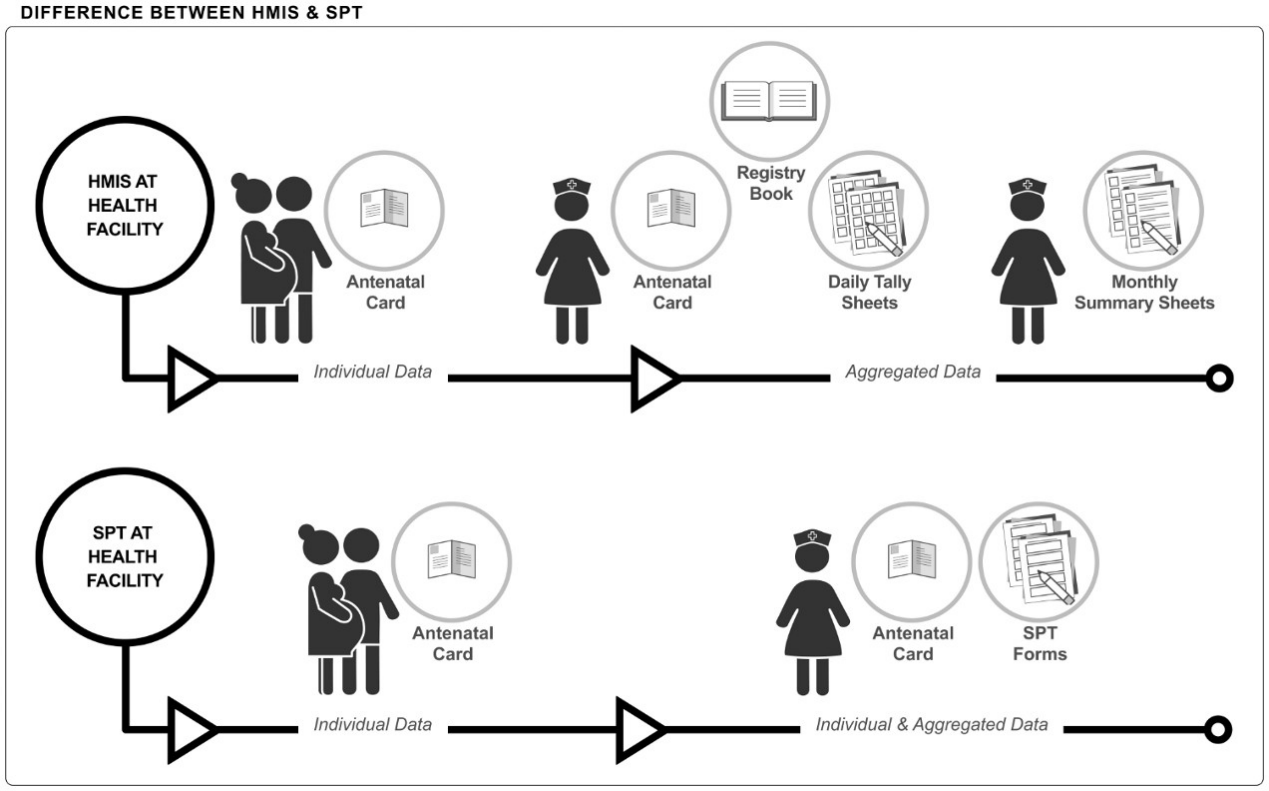
The difference between HMIS and SPT systems at health facility level. HMIS: A nurse documents patient and service information into a woman’s antenatal care card and in a register book. Daily tally sheets and monthly report forms are manually created. The latter are brought to the district headquarter up to 10^th^ of each month. SPT: A nurse documents patient and service information in a woman’s antenatal care card and on one scannable SPT form. Forms are brought to district headquarter in regular intervals.

**Fig 2:**
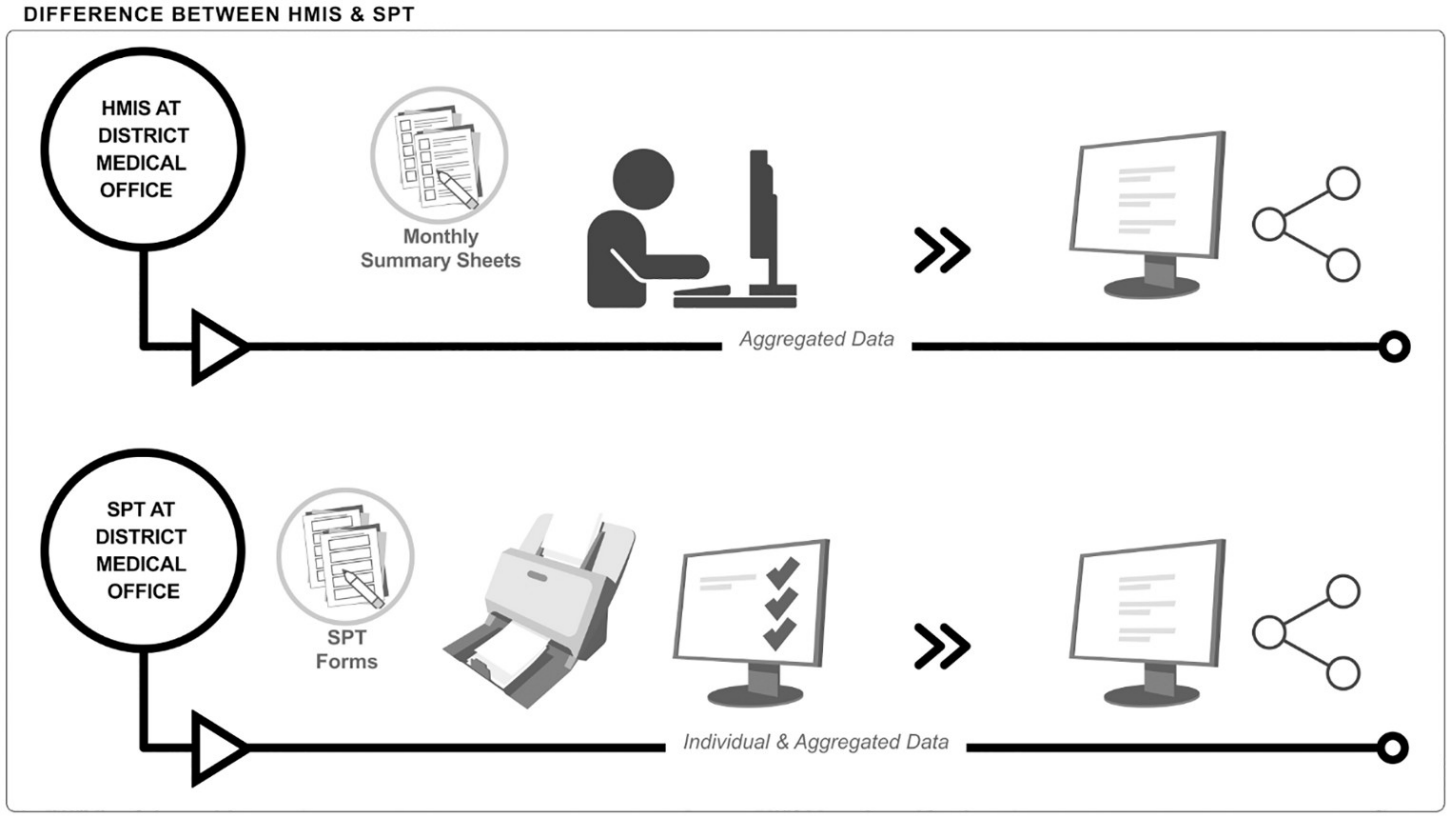
The difference between HMIS and SPT systems at district level. HMIS: Several health care managers manually enter information from monthly report forms of all health facilities into the electronic DHIS2 system. Obvious inconsistencies or errors detected during data entry are followed up by phone or during physical visits where registers are checked. In DHIS2 aggregated data can be viewed from facilities with paper-based data entry (dispensaries) and both individual and aggregated data from fully digitized data entry (many health centres and all hospitals). SPT: One scanning officer scans all forms per facility. Data is automatically read by the software. Inconsistencies or errors are flagged by the system. A data verification officer works on these using the electronic register and phone calls to facilities if needed. Daily tally sheets and monthly summary reports are created automatically. Individual and aggregated data can be viewed.

At present, the electronic *District Health Information System* (DHIS2) is used to collate HMIS data in over 70 countries worldwide [5]. Individual patient data is recorded manually in HMIS paper-based registers commonly known in Kiswahili as *MTUHA (Mfumo wa Taarifa za Uendeshaji Huduma za Afya*), then summarised on monthly report forms in health facilities (Fig 1 below). Aggregated summary data from these reports is entered manually into DHIS2 at district level [6] where various users can view this summary data at different levels of aggregation (Fig 2 below). While the system is well established, incompleteness and inconsistency of data due to calculation and reporting errors have been described [7–10]. Also, digital aggregated data is often not available on time for decision making especially at sub-national level [11]. Another limitation of the present system is the lack of individual-level data making it impossible to construct effective coverage indicators [12].

Digitalization at the first step of HMIS–where data is generated–has the potential to alleviate these problems [13]. *The Smart Paper Technology* (SPT) system uses an innovative software to scan and digitize information from paper forms (hybrid-digital system) and improve the collection of routine health data (Figs 1 and 2 below). These forms, matching HMIS register content for ante-, intrapartum and postnatal care (S1 Fig), completed at facility level, are scanned at district level and generate digital individual patient data as well as aggregated data. In addition to monthly summary reports, the system continuously generates electronic data on a dashboard where both individual and summary data can be viewed. Data inconsistencies are signaled automatically. SPT was first used for documentation of vaccination service data in Uganda and The Gambia [14, 15].

We introduced SPT in one typical district council with 32 primary facilities and one hospital in Southern Tanzania for the continuum of antenatal, intrapartum and postnatal care between May 2019 and June 2020.

Digital innovations need to be relevant to context and user group feedback is important to support implementation and to evaluate potential for scale up [16, 17]. Technology that is not adapted to the context may increase time spent on data generation, negatively affecting service provision and consequently patient safety [18, 19]. End users may fail to integrate the new technology into existing systems with negative effects for sustainability. Taking these recommendations into account, our study aimed to evaluate feasibility, usability and acceptability of SPT to understand why, how and to what extent users engage with SPT and to identify potential positive and negative effects of this newly introduced digital technology. We are reporting our findings and conclusions here using the *Checklist of Mixed Methods Elements* proposed by Fetters et al. [20] and the *Consolidated Criteria for Qualitative Reporting* by Tong et al. [21].

## Methods

### Overall design and conceptual framework

We adopted a convergent parallel design [22] applying a mix of qualitative and quantitative methods grounded in social constructivism [23]. The PRISM framework (*Performance of Routine Information Management*) [24] and *Normalization Process Theory* (NPT) [25] informed our conceptual framework (S2 Fig). NPT applies four constructs to explain how users integrate new technologies into existing routines: i) coherence (“*users assign meaning and utility to a new technology*”) ii) cognitive participation (“*users engage with new technology by their own agency or by assignment*”), iii) collective action (“*users organize and operationalize new technology*”) and iv) reflexive monitoring (“*users evaluate new technology informally or via formalized platforms*”) [25].

Our qualitative study part used focus group discussions (FGDs) with health care providers (HCPs) and in-depth interviews (IDIs) with health care managers exploring SPT feasibility, usability and acceptability. The quantitative time-motion study compared time spent by HCPs on documentation and patient care before and after SPT introduction.

### Setting

The project was conducted in one rural district in Southern Tanzania in cooperation with the Ministry of Health and local government officials at national, regional and district level. The district has a population of 200,000 inhabitants, predominantly subsistence farmers [26, 27]. A phased introduction of SPT took place over 13 months, accompanied by mentoring visits by the project team and remote mentoring and troubleshooting via WhatsApp. HCPs were trained on content and completion of forms. Selected managers at district level received training on dashboard use. Duplicate data entry into both SPT forms and HMIS registers was requested by the ministry throughout the study to maintain routine reporting to national level.

### Sampling strategy and recruitment

Tandahimba district council was purposefully selected because of a pre-existing research programme [28, 29]. From the 33 facilities within the district council, we sampled three public facilities, the only hospital, one health centre and one dispensary to include the three levels of care. Facilities were chosen for accessibility, case load and because they were part of the first scale-up wave.

The qualitative sampling strategy applied purposive sampling for FGDs and IDIs, based on the concept of information power [30]. The participants were nurse-midwives from ante- and postnatal care and labour wards and a few clinicians in-charge who processed data for HMIS and SPT.

For the quantitative time-motion study, we included all HCPs from selected facilities present on observation days. Informed by previous studies [31–33], we calculated that 240 hours of observation were needed before and after SPT introduction (after inflating by factor 1.5 to adjust for clustering) to determine a change in working-time spent on documentation (primary outcome) with 80% power at 5% significance level.

### Data collection

Two data collectors fluent in Kiswahili with previous experience in qualitative research (JB, RU) collected qualitative data in July 2019, one month after SPT introduction (“post-intervention”, four FGDs, six IDIs) and in February 2020, eight months after introduction (“follow-up”, four repeat-FGDs, five repeat-IDIs). Informed by the conceptual framework (S2 Fig) and literature on evaluation of digitalization in health care and HMIS, we developed topic guides [17, 24, 25, 34] (S3 Fig). Tools were pre-tested and further adapted for follow-up data collection to accommodate new themes from first data analysis. FGDs were conducted after working hours and participants travelling from home received compensation of approximately 4 US dollar. IDIs and FGDs lasted on average one hour. FGDs were conducted in facilities, in rooms separate from patient care. IDIs were conducted in participants’ offices. Both were audio-taped after written informed consent and field notes were taken. Frequent summaries were provided to participants during interviews to countercheck information and enhance trustworthiness.

We collected time-motion data at baseline (February and March 2019) and two months after SPT introduction (August 2019). After a 5-days training, two observers per facility with clinical background, shadowed HCPs for their entire shifts, starting from HCPs’ arrival at their consultation desk. Morning and afternoon shifts, as well as weekends, but no night shifts were included. The data collection tool, informed by Pizziferri et al. [35] and initially piloted with health managers, included 141 tasks under 3 categories and 10 sub-categories, to reflect daily tasks of ante-, perinatal and postnatal care (S1 Table). The tool was translated into Kiswahili, programmed into *Open Data Kit* (GetODK. Inc, version 1.2.4.1) and administered on tablets. Pilot observations and feedback sessions conducted during observer training reduced inter-observer variability and ensured that tasks were sufficiently defined and discriminated (task calibration) [36].

All data was anonymized and stored on a password-protected computer. We used a protected cloud at Karolinska Institute for data sharing during joint analysis.

### Data analysis

Qualitative recordings were transcribed ad verbatim without review by participants for logistic reasons. We (RU, UB, JB) applied reflexive thematic analysis [37, 38] with initial inductive and later deductive coding in NVivo 12 pro (QSR International) on Kiswahili transcripts. After initial blinded coding of four transcripts, a coding tree was developed and applied across the data set. Post-intervention and follow-up data sets were analyzed separately to explore changes in feasibility, usability and acceptability throughout the course of the intervention. We then jointly developed sub-categories, categories and themes and applied the conceptual framework for latent analysis. Although we developed themes emerging from primary data, they are inspired by the notion of Greenhalgh and Swinglehust that “*technology shapes humans but is also shaped by human interaction*” [39].

For the time-motion study, we first compared the number and average duration of observed shifts before and after SPT introduction and for potential confounders (level of care, section, cadre & experience) using Fisher exact test. We estimated the *difference in proportion of time spent on individual task categories and sub-categories per average shift* between pre- and post-intervention. We then used multivariable generalised linear models to assess differences in proportion for each task category between pre- and post-SPT implementation adjusting for potential confounders. The category medical doctor was removed from the analysis since there was only one observation for this cadre from the pre-implementation data collection.

### Ethical considerations

We received ethical clearance from the *Institutional Ethical Committee of Ifakara Health Institute* (IHI/RB/No.20 -2018) and *National Institute of Medical Research* (NIMR/HQ/R.8a/Vol.IX/3018) in Tanzania and from the *Ethics Review Board of the Commune of Stockholm* (2019–04022 Gk), Sweden.

We obtained informed written consent from each participant. Observers assessed suitability for observation at each patient encounter according to agreed standards. Patients were informed about the purpose of the observation, the benefits to the population and their right to object.

## Results

### Qualitative results

We report key results from eight FGDs and 11 IDIs. A total of 16 women participated in FGDs but only two men. Three women and three men participated in IDIs (Table 1 below). No medical doctor participated in the study but three non-physician clinicians.

**Table 1:** Participant characteristics.

Method	Men	Women	Cadre
Focus Group Discussions (FGDs) (4 post-intervention, 4 follow-up)	2	16	Nurse-Midwife 9
			Non-Physician Clinician 1
			Assistant Nurse 5
			Medical Aide 3
In-depth Interviews (IDIs) (6 post-intervention, 5 follow-up)	3	3	Nurse-Midwife 3
			Non-Physician Clinician 3

We identified three themes with eight categories, 19 sub-categories, 95 codes (S2 Table). Below we summarize our findings, identifying three main themes i) Technology shaping human interaction, ii) Human interaction shaping the use of technology and iii) Technology and human interaction shaping SPT performance.

### Theme 1 Technology shaping human interaction

This theme explores how technical factors related to SPT influenced i) data processes and interaction with clients and each other (category 1) and ii) HCPs practices regarding data accountability through a mandatory signature on each form (category 2).

#### Category 1 Technical factors influencing data processes

HCPs described how form design led to time-savings: They appreciated that SPT fostered i) reduced writing and ii) simultaneous documentation and patient care.

“*You know*, *with SPT you just tick*. *There is no need to write much*. *You can spend time with your client while ticking the form*

(FGD3 post-intervention)

HCPs discovered ways to reap benefits from form design for documentation AND patient care.

Managers added that the SPT system enabled them to control work performance.

"*With SPT you will identify HCPs quickly [who do not perform]*, *because if they don’t provide services*, *they won’t bring* forms."

(IDI1 follow-up)

Managers realized different benefits from the technology, and these may be perceived as disadvantageous by HCPs.

Managers also saw disadvantages, i.e. regarding low data ownership: i) problematic dashboard access and ii) lack of training in data verification and scanning prevented use of SPT data.

"*As I have said before we would use it [data] if we could generate data that is ours*. *If we could scan*, *at least we could use it*. *But for now*, *to say we have used it is difficult*.”

(IDI5_follow-up)

Technology issues could potentially decrease perceived SPT coherence. The quote above emphasizes the importance of including all user groups to achieve institutionalization of a new technology. The fact that managers could not use SPT data like HMIS data may have affected their views on system coherence, and cognitive participation in the implementation of SPT.

#### Category 2 Technical factors shape health care providers’ practices

HCPs explained how use of SPT forms changed service documentation from i) being a burden and easy to falsify for HMIS, to ii) acknowledging the importance of entering correct data for SPT.

Each SPT form was signed by the respective HCP prompting them to document only what they had done.

"*This shows straight who filled the form because you must sign*… *But those registers… In some*, *pages are torn or gone*. *It is easy to cook up that information*. "

(FGD1 follow-up)

HMIS registers stayed in facilities, so managers could not check these unless travelling to the facility. SPT forms in contrast were brought for scanning to district headquarters and data quality assurance was done during this process indicating how the SPT technological design stimulated change.

### Theme 2 Human interaction shaping the use of technology

Under this theme we report how HCPs’ lack of analytical skills and managers’ views about primary and reported data may have shaped SPT use (category 1) and how the resulting data culture may have disempowered HCPs further to use the data they generated (category 2). We further describe how known organizational challenges, often called health system bottlenecks, influenced the integration and institutionalization of SPT (category 3).

#### Category 1 Health care providers’ capacities shaping the use of technology

Participants proposed that HCPs lacked analytical skills including simple calculation.

“*They do not have enough skills… We tell them to write down their top-ten-diseases from the report and what they get is different to what we have*.”

(IDI2 follow-up)

Although HCPs felt they collected data with SPT more efficiently than previously, the lack of analytic skills limited SPT’s full potential, e.g. for service improvement.

#### Category 2 Data culture shaping data generation and use

Data culture was characterized by managers directing HCPs on data use, who remained recipients of these directives without an active role. HCPs described their task as generating rather than analyzing data.

“*Yeah*, *we just collect it [data] and then we bring it to them [health care managers]*.”

(FGD2 follow-up)

HCPs’ agency to engage with SPT was not substantial enough to facilitate the use of data because they felt disempowered to do so.

Managers nonetheless valued data quality highly for their own work because they were accountable to their superiors.

"*Oh yes*, *these days it’s about data*. *Every work we do*, *we must use data…This is why we emphasize to them [HCP] that what they report needs to be of quality*."

(IDI3 follow-up)

Managers’ emphasis was on reported data, the final product of data processing, rather than primary data, so the system in place for HMIS seemed satisfactory. They may not have seen a need to advocate for using SPT only, nor to change their approach to primary data collection.

#### Category 3 Organizational factors influencing use of SPT

Participants described how known health system bottlenecks negatively impacted implementation of SPT, e.g. lack of i) human resources, ii) transport to distribute forms and iii) supervision.

"*For this [SPT] we have not yet gone for supervision*. *Presently we only know that providers do that work because we have seen the report*. *And you can see once someone runs out of forms*, *they come to ask for new ones*.”

(IDI3_post-intervention)

Health system-related problems known to impact HMIS were also important for SPT implementation. These challenges may have affected HCPs’ and managers’ cognitive participation and collective action related to SPT institutionalization because of failure to i) see its utility within their working environment and ii) implement planned activities to embed SPT. Most of these bottlenecks related to funding not amendable by HCPs nor managers.

The project had set up a WhatsApp group where managers, HCPs and project staff could discuss problems related to the content or logistics of the forms. The virtual nature of the platform may have alleviated some of the challenges regarding supervision. Apart from those tangible benefits, it also created an opportunity for reflexive monitoring of SPT implementation. Still, group composition reflecting organizational hierarchy may have prevented a joint and honest reflection on implementation status, also given the organizational data culture.

### Theme 3: Technology and human interaction shaping SPT performance

Here we explore how technological factors related to SPT and HCPs’ and managers’ efforts or the lack thereof influenced SPT data collection processes (category1), data quality as SPT output (category 2) and the reported impact on other practices, such as patient care (category 3).

#### Category 1 Improved data collection processes

HCPs felt competent using SPT and described creative ways to *improve data processes* by re-distributing staff and using notebooks to track unique identifiers.

"*We have two rooms for service provision here*. *In each room we have now put two people*."

(FGD4 post-intervention)

The degree of cognitive participation and collective action of HCPs as technology users seemed important here to overcome system challenges integrating a new technology. HCPs re-distributed staff despite lack of human resources by finding smart solutions for workflow re-design.

HCPs also described specific quality assurance strategies for SPT, such as i) individual checks against HMIS register, ii) peer-check and iii) checks by immediate superior.

"*Besides that [check by facility in-charge] we do our own check-up*: *If today I have seen six patients*, *my SPT forms should match that number*… *If they do not match*, *you must start checking which patient you omitted and how*."

(FGD3follow-up)

HCPs’ agency and accountability may have shifted from feeling as data producers to controlling the quality of their own data, an opportunity for a positive data culture. Given the challenging environment and prevailing data inconsistencies, it is however likely that quality assurance was often dropped.

#### Category 2 Individual and organizational practices influence SPT output

Improved documentation processes apparently did not lead to improved data quality. Apart from system bottlenecks, HCPs explained how duplicate data entry contributed to this. They admitted that data was missing but still perceived their data quality assurance measures worked.

“*…We complete the forms and then we enter the data [into HMIS registers] instantly*. *Sometimes you may fail to complete the register*. *When we write the report*, *we may miss some [women]*”.

(FGD4 follow-up)

The contradiction between data discrepancies and HCPs’ descriptions of data quality assurance may be an indication for the degree of previous HMIS data fabrication, only known to HCPs since facility registers were concerned. It may also highlight HCPs motivation to continue using SPT because they clearly saw its benefits.

Managers mainly emphasized HCPs’ i) lack of commitment, ii) forgetfulness and iii) duplicate data entry as causes for data discrepancies.

“*They have not reached completeness that is true*. *They may have forgotten to enter all [clients] and this is a question of commitment*. *Once this has been improved*, *everything will arrange itself*.”

(IDI5 post-intervention)

Managers put more emphasis on human factors, such as HCPs attitude and forgetfulness. This may imply a perceived lower organizational responsibility for data quality. Managers may also have preferred duplicate data entry to maintain their ways of working, being less ready for change than HCPs.

#### Category 3 SPT use influences other processes

HCPs described how their efforts to integrate SPT in their daily work, led to re-organized service provision and teamwork.

"*We have organized ourselves well to ensure that everything needed for SPT or maternal and child health is available*. *We had manual BP machines*, *now we have automatic*, *Urine [sticks] are available*… *we make sure everything is there to provide services at the right time so things can move forward*. "

(FGD2follow-up)

Participants explained how SPT use promoted accountability for patient care and indirectly also improved their working environment. This reported accountability may link to the mandatory signature and to another human being interacting with it during scanning and quality assurance. This finding emphasizes the complexity of human-technology interaction and the need for a holistic evaluation when introducing digital technology.

### Quantitative results: Time-Motion-Study

We observed a total of 273.3 hours before and 224.0 hours after SPT introduction in three pilot facilities. A total of 3,354 tasks was observed pre-and 3,803 post-intervention.

An average observed shift ranged from 0.3 hours to 19.7 hours (median (IQR) 5.4 (4.4–6.1) hours), with no differences between baseline (median (IQR) 5.5 (4.0–6.0) hours) and follow-up assessment (median (IQR) 5.3 (4.4–6.1) min) (S3 Table).

We saw no difference in the median duration of observed shifts across level of care and professional cadre during pre- and post-intervention assessments. We documented, however, more antenatal care services during baseline in comparison to post-intervention.

We observed that around 10% of an average shift was spent on *HMIS documentation*: 11.6% (95% CI 7.7, 15.5) before and 9.8%, (95% CI 7.4, 12.1) after SPT implementation (p 0.627) (Table 2 below). *SPT documentation* only added 3% of time spent to *overall documentation* per average shift (CI 1.9, 4.1). The proportion of time spent on *overall documentation* per average shift did not differ (27.0% (CI 22.6, 31.4) and 26.4% (CI 22.7, 30.7); adjusted p = 0.763) (Table 2 below).

**Table 2:** Proportion of time spent per average shift by task sub-categories and categories.

Task sub-categories and categories	Proportion of average shift (%)before SPT implementation(n = 50)	95% CI	Proportion of average shift (%)after SPT implementation(n = 43)	95% CI	p-value from generalized linear model	Adjusted p-value from multivariable model^#^
**Overall documentation (+/- SPT admin, indirect care)** ^a^	**27.0**	[**22.6, 31.4**]	**26.4**	[**22.7, 30.2**]	**0.838**	**0.763**
HMIS documentation (non-SPT)	11.6	[7.7, 15.5]	9.8	[7.4, 12.1]	0.401	0.627
SPT documentation	-	-	3.0	[1.9, 4.1]	-	-
Indirect care	15.4	[11.8, 19.1]	13.7	[10.8, 16.6]	0.444	0.530
**Direct patient care (antenatal, postnatal, labour, outpatient care** ^c^ **)**	**26.9**	[**22.4, 31.5**]	**37.1**	[**32.3, 41.9**]	**0.002** *	**0.001** **
Antenatal care	11.4	[7.0, 15.8]	14.9	[9.0, 20.9]	0.324	<0.001**
Care during labour	7.5	[3.9, 11.1]	10.4	[5.6, 15.1]	0.324	0.949
Postnatal care	5.3	[2.3, 8.4]	7.7	[4.2, 11.2]	0.310	0.905
Outpatient care	2.7	[0.2, 5.3]	4.2	[0.1, 7.5]	0.490	0.206
**Other tasks (walking, waiting, personal, miscellaneous)** ^b^	**46.0**	[**39.4, 52.7**]	**36.4**	[**29.8, 43.0**]	**0.039** *	**0.007** **
Miscellaneous	12.3	[7.2, 17.4]	10.3	[7.2, 13.4]	0.475	0.317
Personal	19.5	[12.3, 26.6]	14.5	[9.8, 19.3]	0.231	0.160
Waiting	7.9	[4.1, 11.7]	5.5	[3.3, 7.7]	0.247	0.790
Walking	6.3	[4.1, 8.6]	6.1	[4.1, 8.1]	0.879	0.078

*Statistical significance in linear model,

** Statistical significance in multivariable model,

^#^ Variables used for adjustment were “level of care”, department”, “cadre” and “professional experience”,

^a^ Individual tasks e.g. documentation within HMIS registers, SPT-related documentation and indirect care (e.g. organizing referral, communicating with relatives, filling the partograph, filling antenatal care cards etc.),

^b^ four sub-categories, i.e. miscellaneous (e.g. observer unsure about task, shift hand over, observer personal hygiene, non-medical reading etc.), personal (e.g. personal phone calls, personal hygiene, on break), walking and waiting.

^c^ outpatient care was only performed by staff from ante-/postnatal and labour wards at dispensary level and SPT was not introduced at outpatient care

We observed that time spent on the aggregated category *direct patient care* increased from 26.9% (22.4, 31.5) per average shift at baseline to 37.1% (CI 32.3, 41.9) at post-intervention (adjusted p 0.001) (Table 2 below). We saw no significant change in time per average shift spent on individual task sub-categories related to *patient care* except for *antenatal care*. Here we noted an increase in proportion of time spent from 11.4% (CI 7.0, 15.8) to 14.9% per average shift (CI 9.0, 20.9) after adjustment (p <0.001) (Table 2 below).

We observed a significant change in the aggregated category *other tasks*, which decreased from 46.0% (CI 39.4, 52.7) to 36.4% (CI 29.8, 43.0) (adjusted p 0.007) per average shift after SPT introduction. However, changes within the respective sub-categories did not reach statistical significance (Table 2 below).

## Discussion

In summary our findings indicate that HCPs and their managers perceived SPT as time-saving, functional and well-aligned to pre-existing work- and documentation-processes, supporting its acceptability and usefulness within the Tanzanian context. Quantitative findings suggest feasibility related to time-savings without negative impact on patient care. Managers had concerns about data ownership and quality and consequently did not fully engage with SPT. Known health system bottlenecks may have impaired integration. Participants described a data culture disempowering HCPs regarding data analysis and use, but depicted HCPs’ agency for embedding SPT despite challenges. They also recounted a secondary effect of SPT design on HCPs’ accountability for data and service provision.

### Technology shaping human interaction

SPT shaped user action and interaction directly which may have contributed to its acceptability, feasibility and usability: HCPs saw its coherence early in implementation and this enhanced their cognitive participation in collectively embedding it [25]. These findings on SPT acceptability are in line with a study evaluating SPT for child vaccination services in Uganda [33].

Managers acknowledged benefits, but difficult dashboard access impeded their SPT data use apart from monthly report data. This important technical factor may have impacted on managers’ engagement decreasing their efforts to i) integrate SPT in organizational practice, ii) support HCPs’ collective action and iii) engage in reflexive monitoring [25]. Other research confirms data access as an important determinant of digital technology implementation [40–42].

Research introducing digital patient records pointed to increased time spent on documentation: Were et al. reported reduced provider time spent with clients in Uganda and argued that HCPs being unfamiliar with the technology took more time for documentation after clients had left [31]. Similarly, Rotich et al. described increased time for patient registration in Kenya [32]. In contrast, we report no significant difference in time spent on overall documentation per average shift before and after SPT introduction (27.0% to 26.4%; adjusted p 0.763) without negative impact on time spent on patient care (26.9% before, 37.1% after SPT introduction; adjusted p = 0.001). SPT uses paper forms based on familiar HMIS content. This may partly explain these differences. Our findings confirm other SPT evaluations. The study in Uganda found a neglectable 24 seconds increase in documentation time per immunization service [33]. A similar study in The Gambia found a reduction by 16 minutes per child for SPT [15].

SPT technology also indirectly impacted HCP practices: The mandatory signature reportedly fostered feelings of accountability. Although individuals could be associated to HMIS data previously, our participants did not perceive this as a similar trigger towards data quality. Studies from Tanzania and comparable settings describe HCPs’ HMIS data manipulation related to organizational pressure [9, 43–45] and report low data accountability for data [24].

### Human interaction shaping the use of technology

Our evaluation indicated that data use remained weak. SPT decreases calculation errors through automated tallying, but it cannot address users’ abilities to analyse and interpret data. Research suggests appropriate skills are essential for data use [46–48] and that projects introducing technology should include capacity building to improve analysis skills [49–51].

The setting’s data culture promoted analysis by managers. Lippefeld advocates for managerial role models for a positive data culture [52] but studies from several countries report managerial complacency to data manipulation [43–45]. Our participants did not mention this, but managers’ emphasis on the summary report may indicate similar complacency.

Other organizational factors may have influenced feasibility and usability of the SPT system. Lack of human resources and supervision have been documented as barriers to HMIS data quality in Tanzania [9, 10, 47]. Our quantitative findings do not support qualitative results regarding HCPs’ workload influencing SPT data entry: Time spent on other tasks, e.g. private tasks, took up 36.4% per average shift after SPT introduction. This finding is in line with the study by Were et al. [31]. In both studies, HCPs seemed to have buffer time for tasks not directly related to documentation or patient care.

Other studies report on the importance of supportive leadership and supervision for integration of digital technology and data use [46, 47, 53, 54] and managers’ supervisory activity concerning SPT may reflect their cognitive participation in the project.

### Technology and human interaction shaping SPT processes and performance

HCPs assigned positive meaning to SPT and improved data collection processes and teamwork despite a challenging environment. This underscores the importance of coherence and cognitive participation for the introduction of technology [55, 56].

This process improvement did not lead to improved outputs regarding data quality although participants lauded the timely availability of SPT data. Reasons for the reported data discrepancy may be found in i) the health system’s organizational culture regarding health data [9, 10, 45, 57], ii) the environment of data collection [47] and iii) duplicate data entry [18].

Our quantitative results show no difference in time spent on overall documentation after SPT introduction despite duplicate entry. Time spent on SPT documentation was only 3.0% per average shift and on HMIS documentation 9.8%, suggesting SPT, once introduced as the only documentation system, could potentially save time. Our participants reported in contrast that data entry may have been incomplete for both HMIS and SPT and HCPs may have prioritized the official HMIS, contrary to their reports of completing SPT first.

SPT did not directly improve data-related outcomes such as data analysis or use. The reported changes in accountability may be an indication that organizational change may still be possible by empowering HCPs to re-design their data processing environment [48, 52] similarly to how they improved their care environment.

**Fig 3:**
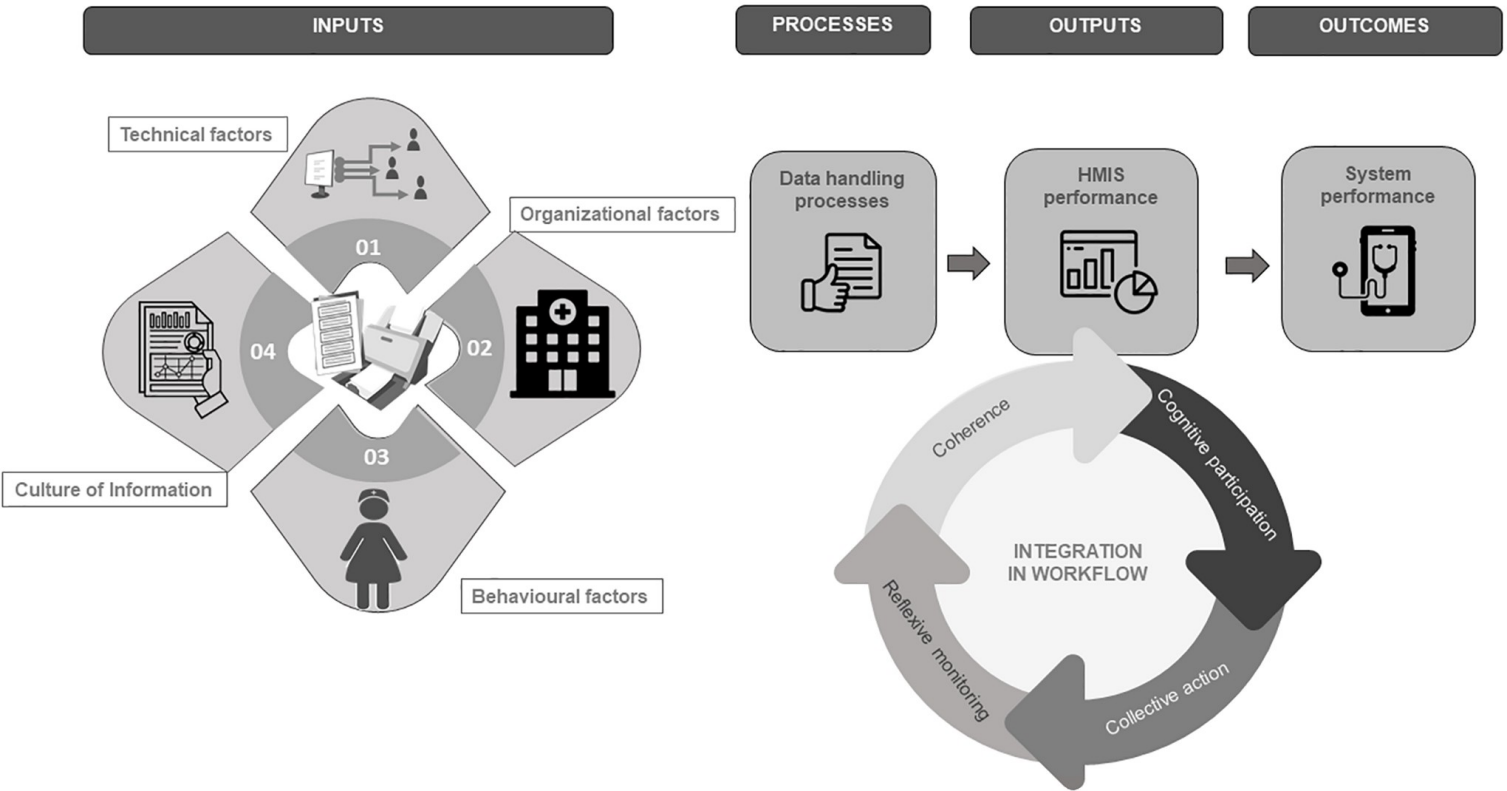
Factors influencing the use of digital technology to impact documentation processes, outputs and outcome.

Our conceptual framework (Fig 3) supported generating knowledge on how SPT technology may have shaped human (inter)action and how this (inter)action may have shaped the use of this technology to influence processes, outputs and outcome.

### Strengths and limitations

Our mixed-methods design has strengths, allowing for triangulation, which is distinctly different from most other studies assessing user acceptance [31] quantitatively. In addition, our design and conceptual framework allowed the evaluation of the entire system of health data processing and its determinants.

Our study also faces limitations. First, we conducted the time-motion study in three out of 33 health facilities only. The observed change in time spent on documentation was much smaller than anticipated, reducing study power. This may have influenced findings for sub-categories because differences in proportion for individual sub-categories became too small to reach significance (Table 2). The study did not include an investigation of time spent on further data processing at district level. No data was collected on patient load during observation.

Our Observers changed between data collections. This may have influenced the number of individual tasks recorded due to different experience of both groups. However, we kept inter-observer variability low by including practice observations with feedback as described in our methods section and by Zheng et al. [36]. Observation may have introduced a Hawthorne effect, prompting HCPs to increase their documentation or care efforts.

Male voices were underrepresented in FGDs. The aim of our study was to generate knowledge about acceptability, feasibility and usability of SPT, which is paper-based at data-entry. We believe that our participants had sufficiently broad knowledge on health data processes to create adequate information power. Further research aiming to understand gender differences in collection and use of digital data would benefit from a theoretical sampling approach with deliberate gender balance.

## Conclusion

Our results indicate that it is feasible to integrate a digital-hybrid system like SPT in resource-constraint settings such as rural Tanzania with good acceptability and usability for primary users and without negative impact on overall documentation and time spent on patient care. Organizational culture and practices play an important part in the institutionalization of new digital technologies. It is important to engage with users at all levels of the health care system in designing and implementation of new technologies to foster adequate degrees of motivation and action to support routine health data collection.

## Supporting information

S1 Fig SPT forms. (PDF)

S2 Fig Conceptual framework. (TIF)

S3 Fig Topic guide focus group discussion post implementation (English version). (DOCX)

S1 Table Task list for programming. (DOCX)

S2 Table Overview of themes and categories. (DOCX)

S3 Table Distribution of shifts observed before/after SPT introduction. (DOCX)

## Abbreviations

DHIS2: District Health Information System 2
FGD: Focus Group Discussion
HCP: Health Care Provider
HMIS: Health Management Information System
IDI: In-depth Interview
NPT: Normalization Process Theory
PRISM: Performance of Routine Health Information Systems Management
SPT: Smart Paper Technology
