# Supplementary material for: Feasibility, usability and acceptability of a novel digital hybrid-system for reporting of routine maternal health information in Southern Tanzania: A mixed-methods study
Source: PLOS Glob Public Health. 2023 Jan 12;3(1):e0000972. doi: 10.1371/journal.pgph.0000972 (PMC10021923; doi:10.1371/journal.pgph.0000972)
Supplement: S1 Fig — (PDF) [file pgph.0000972.s001.pdf]

REGISTRATION FORM

Health facility DEMO Date day - month

01 1 02

Identification number **000-0092**

Name Partner name

Age Height (cm) Local government chairperson

Village Street or/Sub-village Phone number

Pregnancy history

Previous CS ○ Age of youngest child

Number of:

Miscarriages/Abortions Newborn deaths (first week)

Live births Stillbirths Children alive

Current pregnancy

Duration of pregnancy (weeks)

day month

Date of last menstrual period -

Estimated date of delivery -

Identification number **000-0101**

Name Partner name

Age Height (cm) Local government chairperson

Village Street or/Sub-village Phone number

Pregnancy history

Previous CS ○ Age of youngest child

Number of:

Miscarriages/Abortions Newborn deaths (first week)

Live births Stillbirths Children alive

Current pregnancy

Duration of pregnancy (weeks)

day month

Date of last menstrual period -

Estimated date of delivery -

01 1 02

Shifo Karolinska Institutet IFAKARA HEALTH INSTITUTE

Ministry of Health, Community Development, Gender, Elderly and Children, The United Republic of Tanzania

DEMO

REGISTRATION FORM

01 2 02

Identification number **000-0117**

Name | Partner name

Age | Height (cm) | Local government chairperson

Village | Street or/Sub-village | Phone number

Pregnancy history

Previous CS ◯ | Age of youngest child

Number of:

Miscarriages/Abortions | Newborn deaths (first week)

Live births | Stillbirths | Children alive

Current pregnancy

Duration of pregnancy (weeks)

day – month

Date of last menstrual period _

Estimated date of delivery _

Identification number **000-0125**

Name | Partner name

Age | Height (cm) | Local government chairperson

Village | Street or/Sub-village | Phone number

Pregnancy history

Previous CS ◯ | Age of youngest child

Number of:

Miscarriages/Abortions | Newborn deaths (first week)

Live births | Stillbirths | Children alive

Current pregnancy

Duration of pregnancy (weeks)

day – month

Date of last menstrual period _

Estimated date of delivery _

Shifo Karolinska Institutet IFAKARA HEALTH INSTITUTE

Ministry of Health, Community Development, Gender, Elderly and Children, The United Republic of Tanzania

DEMO

REGISTRATION UPDATE FORM

Health facility DEMO — Date (day – month)

02 1 02

Identification number –

Name | Partner name

Age | Height (cm) | Local government chairperson

Village | Street or/Sub-village | Phone number

Pregnancy history

Previous CS ◯ | Age of youngest child

Number of:

Miscarriages/Abortions | Newborn deaths (first week)

Live births | Stillbirths | Children alive

Current pregnancy

Duration of pregnancy (weeks)

Date of last menstrual period (day – month)

Estimated date of delivery (day – month)

Identification number –

Name | Partner name

Age | Height (cm) | Local government chairperson

Village | Street or/Sub-village | Phone number

Pregnancy history

Previous CS ◯ | Age of youngest child

Number of:

Miscarriages/Abortions | Newborn deaths (first week)

Live births | Stillbirths | Children alive

Current pregnancy

Duration of pregnancy (weeks)

Date of last menstrual period (day – month)

Estimated date of delivery (day – month)

02 1 02

DEMO

Shifo Karolinska Institutet IFAKARA HEALTH INSTITUTE

Ministry of Health, Community Development, Gender, Elderly and Children, The United Republic of Tanzania

REGISTRATION UPDATE FORM

02 2 02

Identification number ___ – ___

Name | Partner name
Age | Height (cm) | Local government chairperson
Village | Street or/Sub-village | Phone number

Pregnancy history

Previous CS ◯ Age of youngest child

Number of:

Miscarriages/Abortions | Newborn deaths (first week)
Live births | Stillbirths | Children alive

Current pregnancy

Duration of pregnancy (weeks)

day – month

Date of last menstrual period –
Estimated date of delivery –

Identification number ___ – ___

Name | Partner name
Age | Height (cm) | Local government chairperson
Village | Street or/Sub-village | Phone number

Pregnancy history

Previous CS ◯ Age of youngest child

Number of:

Miscarriages/Abortions | Newborn deaths (first week)
Live births | Stillbirths | Children alive

Current pregnancy

Duration of pregnancy (weeks)

day – month

Date of last menstrual period –
Estimated date of delivery –

Shifo Karolinska Institutet IFAKARA HEALTH INSTITUTE

Ministry of Health, Community Development, Gender, Elderly and Children, The United Republic of Tanzania

DEMO

ANTENATAL FORM

Health facility DEMO

Date (day) ___ – (month) ___

03 1 02

Identification number ___ – ___

Visit number: 1 ○ 2 ○ 3 ○ 4 ○ 5+ ○ Has TT card ○ TT1 ○ TT2+ ○

Test results at current ANC visit

BP ___ / ___ Hb ___ , ___

Urinalysis: Sugar ○ Protein ○ | Blood grouping and cross-match done ○

Syphilis: *Woman:* Positive ○ Negative ○ Treated ○
Partner: Positive ○ Negative ○ Treated ○

Other STI: *Woman:* Positive ○ Treated ○
Partner: Positive ○ Treated ○

Repeated visits: No weight gain ○ Vaginal bleeding ○ Bad posture for baby ○ TB ○

PMTCT

	Woman	*Partner*
Previous HIV infection	○	○
Received counselling before testing	○	○
Tested for HIV at current ANC visit	○	○
Tested positive	○	○
Counselled after testing	○	○
Advice on infant feeding	○	
Referred to CTC	○	

Malaria

mRDT/BS: Positive ○ Negative ○ | LLIN ○ | *IPT:* 1 ○ 2 ○ 3+ ○

Supplements provided: Albendazole/Mebendazole ○

Iron ○ Folic acid ○ IFA ○ Number of capsules ___

Referral given ○ Facility referred from ___

Referred to ___

Reason for referral ___

Comments ___

03 1 02

Shifo Karolinska Institutet IFAKARA HEALTH INSTITUTE

Ministry of Health, Community Development, Gender, Elderly and Children, The United Republic of Tanzania

DEMO

ANTENATAL FORM

03 2 02

Identification number ___ – ____

Visit number: 1 ○ 2 ○ 3 ○ 4 ○ 5+ ○ Has TT card ○ TT1 ○ TT2+ ○

Test results at current ANC visit

BP ___ / ___ Hb __ , _

Urinalysis: Sugar ○ Protein ○ | Blood grouping and cross-match done ○

Syphilis: *Woman:* Positive ○ Negative ○ Treated ○ | ***Other STI:*** *Woman:* Positive ○ Treated ○

Partner: Positive ○ Negative ○ Treated ○ | *Partner:* Positive ○ Treated ○

Repeated visits: No weight gain ○ Vaginal bleeding ○ Bad posture for baby ○ TB ○

PMTCT

	Woman	*Partner*
Previous HIV infection	○	○
Received counselling before testing	○	○
Tested for HIV at current ANC visit	○	○
Tested positive	○	○
Counselled after testing	○	○
Advice on infant feeding	○	
Referred to CTC	○	

Malaria

mRDT/BS: Positive ○ Negative ○ | LLIN ○ | *IPT:* 1 ○ 2 ○ 3+ ○

Supplements provided: Albendazole/Mebendazole ○

Iron ○ Folic acid ○ IFA ○ Number of capsules __

Referral given ○ Facility referred from

Referred to

Reason for referral

Comments

Health worker name Signature

03 2 02

Shifo Karolinska Institutet IFAKARA HEALTH INSTITUTE

Ministry of Health, Community Development, Gender, Elderly and Children, The United Republic of Tanzania

DEMO

LABOUR FORM

Health facility DEMO

Date (day – month): ____ – ____

04 1 02

Identification number ____ – ____

Name ____

	day	month	time
Admission:			
Delivery:			

Labour duration over 12 hours ○

Place of delivery

Health facility ○ BBA ○ TBA ○ Home without TBA ○

Companion at birth ○

Mode of delivery

Spontaneous ○ Vacuum ○ Other ○

CS ○ Breech ○

Cadre of birth attendant MO ○ AMO ○ CO/ACO ○ EN ○ RN ○ MA ○

Birth outcome: Live birth ○ FSB ○ MSB ○ Multiple birth ○ *For multiple births, fill in another labour form with mother's ID and information for other newborns*

Sex of baby: Female ○ Male ○ ***Apgar score:*** 1 min ____ 5 min ____ Breastfeeding within 1 hour ○

Care of the baby No intervention ○ Stimulation ○ Bag and mask ○ Suction ○ Birth weight (g) ____

Labour complications

No complications ○ Sepsis ○ APH ○ Chest pain ○ PPH ○ Maternal exhaustion ○

3rd degree tear ○ Anemia ○ High blood pressure ○ Retained placenta ○ PROM ○

HIV (III or IV) ○ Malaria ○ Obstructed labour ○ Ruptured uterus ○ FGM ○

Before delivery Pre-eclampsia ○ Eclampsia ○

During/within 24h: Pre-eclampsia ○ Eclampsia ○

AMTSL: Oxytocine ○ Misoprostol ○ Ergometrine ○ Controlled cord traction ○ Fundal pressure ○

EmOC: No intervention ○ Blood transfusion ○ Anti-hypertensive drug ○ Hysterectomy ○ Uterotonics ○

Uterine balloon tamponade ○ MVA/D&C ○ Misoprostol ○ Antibiotics ○

iv-Magnesium ○ 3rd/4th degree tear repair ○ CS/Laparotomy ○ Manual removal of placenta ○

PMTCT:

Previous HIV infection: Positive ○ Negative ○ Unknown ○

HIV test result during/after delivery: Positive ○ Negative ○ Unknown ○

Discharge information

Feeding

Supplements ○ Exclusive breastfeeding ○

Received ARV prophylaxis ○ Name of ARV ____

	day	month	cause of death
Maternal death:			
Newborn death:			

Referrals: Mother ○ Baby ○ Referral for ARV at CTC ○ Referred to ____ Referral reason ____

Health worker name ____ Signature ____

04 1 02

Shifo Karolinska Institutet IFAKARA HEALTH INSTITUTE

Ministry of Health, Community Development, Gender, Elderly and Children, The United Republic of Tanzania

DEMO

LABOUR FORM

04 2 02

Identification number ____ – ____

Name

	day	month	time
Admission:		–	
Delivery:		–	

Labour duration over 12 hours ○

Place of delivery

Health facility ○ BBA ○ TBA ○ Home without TBA ○

Companion at birth ○

Mode of delivery

Spontaneous ○ Vacuum ○ Other ○

CS ○ Breech ○

Cadre of birth attendant MO ○ AMO ○ CO/ACO ○ EN ○ RN ○ MA ○

Birth outcome: Live birth ○ FSB ○ MSB ○ Multiple birth ○ *For multiple births, fill in another labour form with mother's ID and information for other newborns*

Sex of baby: Female ○ Male ○

Apgar score: 1 min ____ 5 min ____

Breastfeeding within 1 hour ○

Care of the baby No intervention ○ Stimulation ○ Bag and mask ○ Suction ○

Birth weight (g) ____

Matatizo na Matokeo ya Uzazi

No complications ○ Sepsis ○ APH ○ Chest pain ○ PPH ○ Maternal exhaustion ○

3rd degree tear ○ Anemia ○ High blood pressure ○ Retained placenta ○ PROM ○

HIV (III or IV) ○ Malaria ○ Obstructed labour ○ Ruptured uterus ○ FGM ○

Before delivery: Pre-eclampsia ○ Eclampsia ○

During/within 24h: Pre-eclampsia ○ Eclampsia ○

AMTSL: Oxytocine ○ Misoprostol ○

Ergometrine ○ Controlled cord traction ○ Fundal pressure ○

EmOC: No intervention ○ Blood transfusion ○ Anti-hypertensive drug ○ Hysterectomy ○ Uterotonics ○

Uterine balloon tamponade ○ MVA/D&C ○ Misoprostol ○ Antibiotics ○

iv-Magnesium ○ 3rd/4th degree tear repair ○ CS/Laparotomy ○ Manual removal of placenta ○

PMTCT:

Previous HIV infection: Positive ○ Negative ○ Unknown ○

HIV test result during/after delivery: Positive ○ Negative ○ Unknown ○

Discharge information

Feeding

Supplements ○ Exclusive breastfeeding ○

Received ARV prophylaxis ○ Name of ARV ____

	day	month	cause of death
Maternal death:		–	
Newborn death:		–	

Referrals: Mother ○ Baby ○ Referral for ARV at CTC ○ Referred to ____ Referral reason ____

Health worker name ____ Signature ____

04 2 02

Shifo Karolinska Institutet IFAKARA HEALTH INSTITUTE

Ministry of Health, Community Development, Gender, Elderly and Children, The United Republic of Tanzania

POSTNATAL FORM

Health facility DEMO   Date day – month

05 1 02

Identification number –

Maternal death ◯ Newborn death ◯ BP (mmHg) / Hb (g/dl) ,

Breasts
Normal ◯
Cracked nipples ◯
Mastitis ◯
Abscess ◯

HIV test at postnatal ward: Positive ◯ Negative ◯

Pre-eclampsia 24h after delivery ◯
Eclampsia 24h after delivery ◯

Uterus
Normal ◯ Pain ◯
Not well contracted ◯

Lochia
Normal ◯
Abnormal colour ◯

Fistula: Yes ◯ No ◯

Mental status: Normal ◯ Abnormal ◯

Perineum: Healed ◯ Not healed ◯ Infection ◯

Family planning services provided
Counselling ◯ FP method given ◯
Information materials ◯ Referral for FP method ◯

Supplements given
Iron ◯ Folic acid ◯ IFA ◯ Number of tablets
Vitamin A ◯ Number of tablets

Mtoto

For multiple births, fill in another form with mother's ID and information for the baby

Temp (°C) BCG ◯ OPV ◯ Vitamin K ◯ Weight (g) Hb (g/dl) , Pallor (palms) ◯ KMC initiated ◯

Infections: No sign of infection ◯ Umbilical cord ◯ Skin ◯ Oral ◯ Eye ◯ Jaundice ◯ Sepsis ◯

PMTCT: *HIV test result:* Positive ◯ Negative ◯ ARV given ◯ Name of ARV Duration (weeks)

Feeding: Exclusive ◯ Replacement ◯ Mixed ◯

Rufaa

Referral from Referral to Reason

05 1 02

Shifo Karolinska Institutet IFAKARA HEALTH INSTITUTE

Ministry of Health, Community Development, Gender, Elderly and Children, The United Republic of Tanzania

POSTNATAL FORM

05 2 02

Identification number ___ – ___

Maternal death ○ Newborn death ○ BP (mmHg) ___ / ___ Hb (g/dl) ___,___

Breasts

Normal ○

Cracked nipples ○

Mastitis ○

Abscess ○

HIV test at postnatal ward: Positive ○ Negative ○

Pre-eclampsia 24h after delivery ○

Eclampsia 24h after delivery ○

Fistula: Yes ○ No ○

Mental status: Normal ○ Abnormal ○

Uterus

Normal ○ Pain ○

Not well contracted ○

Lochia

Normal ○

Abnormal colour ○

Perineum: Healed ○ Not healed ○ Infection ○

Family planning services provided

Counselling ○ FP method given ○

Information materials ○ Referral for FP method ○

Supplements given

Iron ○ Folic acid ○ IFA ○ Number of tablets ___

Vitamin A ○ Number of tablets ___

Mtoto

For multiple births, fill in another form with mother's ID and information for the baby

Temp (°C) ___ BCG ○ OPV ○ Vitamin K ○ Weight (g) ___ Hb (g/dl) ___,___ Pallor (palms) ○ KMC initiated ○

Infections: No sign of infection ○ Umbilical cord ○ Skin ○ Oral ○ Eye ○ Jaundice ○ Sepsis ○

PMTCT: *HIV test result:* Positive ○ Negative ○ ARV given ○ Name of ARV ___ Duration (weeks) ___

Feeding: Exclusive ○ Replacement ○ Mixed ○

Rufaa

Referral from ___ Referral to ___ Reason ___

Health worker name ___ Signature ___

05 2 02

DEMO
