# Supplementary figures and images for: Feasibility, usability and acceptability of a novel digital hybrid-system for reporting of routine maternal health information in Southern Tanzania: A mixed-methods study
Source: PLOS Glob Public Health. 2023 Jan 12;3(1):e0000972. doi: 10.1371/journal.pgph.0000972 (PMC10021923; doi:10.1371/journal.pgph.0000972)

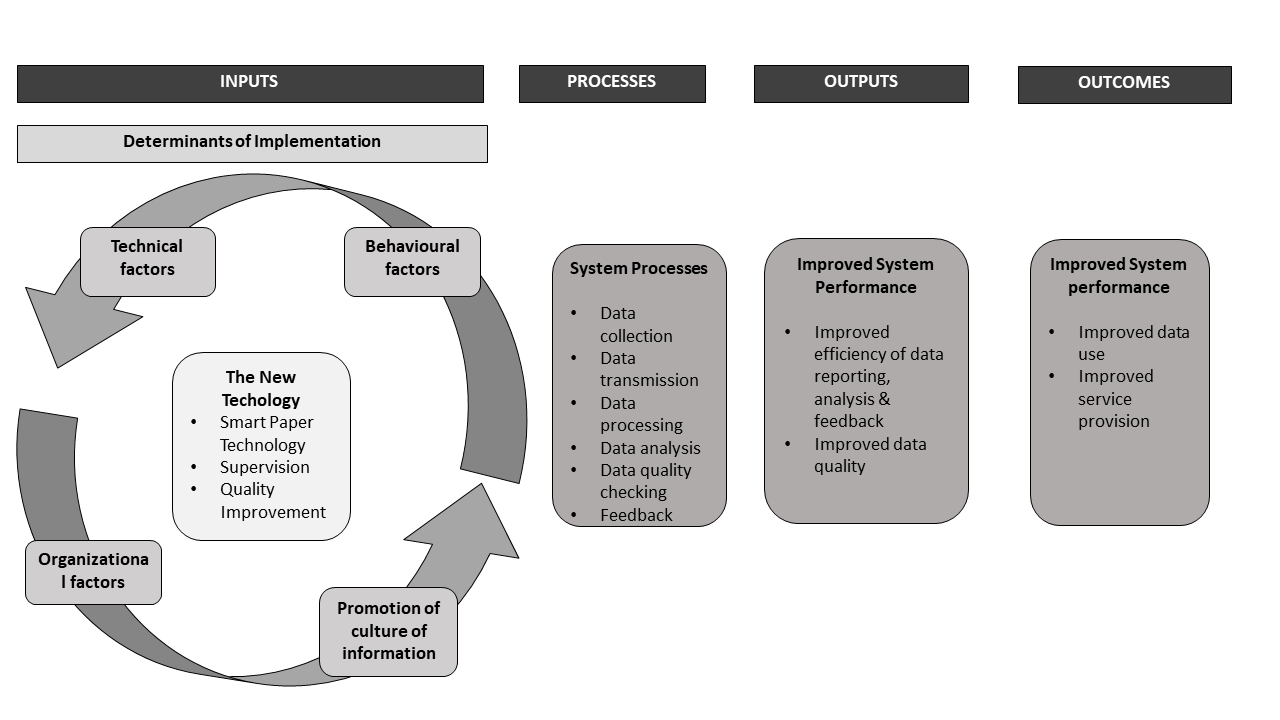

Supplement: S2 Fig — (TIF) [file pgph.0000972.s002.tif]
