# Supplementary material for: Feasibility, usability and acceptability of a novel digital hybrid-system for reporting of routine maternal health information in Southern Tanzania: A mixed-methods study
Source: PLOS Glob Public Health. 2023 Jan 12;3(1):e0000972. doi: 10.1371/journal.pgph.0000972 (PMC10021923; doi:10.1371/journal.pgph.0000972)
Supplement: S1 Table — (DOCX) [file pgph.0000972.s004.docx]

**S4_Table 1: Task list used for programming**

| categories | 1 | Personal |
| --- | --- | --- |
| categories | 2 | Administrative (Hospital) |
| categories | 3 | Patient care labor room (direct care) |
| categories | 4 | Direct patient care ANC |
| categories | 5 | indirect patient care ANC |
| categories | 6 | waiting |
| categories | 7 | Walking |
| categories | 8 | Serving OPD patients |
| categories | 9 | PNC patient care |
| categories | 10 | Miscellaneous |
|  |  |  |
| personaltsk | 1 | Computer: Email or other (eg browsing) personal |
| personaltsk | 2 | Looking/Waiting: Personal |
| personaltsk | 3 | Miscellaneous: Eating/Drinking/Idle |
| personaltsk | 4 | Miscellaneous: On break |
| personaltsk | 5 | Phone: Personal |
| personaltsk | 6 | Miscellaneous: rest room |
| personaltsk | 7 | Talking: Personal |
|  |  |  |
|  |  |  |
| hosp_admintsk | 1 | Filling patient record in register |
| hosp_admintsk | 2 | Looking for patient record in register |
| hosp_admintsk | 3 | Entering patient record in register |
| hosp_admintsk | 4 | Retriving patient record in register |
| hosp_admintsk | 5 | Reffiling new card |
| hosp_admintsk | 6 | Preparing Taly sheet |
| hosp_admintsk | 7 | Preparint monthly summary |
| hosp_admintsk | 8 | Preparing other report (eg PMTCT) |
| hosp_admintsk | 9 | SPT:Filling registration form |
| hosp_admintsk | 10 | SPT:Filling registration update form |
| hosp_admintsk | 11 | SPT:Filling visit form (PNC ANC Labor forms) |
| hosp_admintsk | 12 | SPT:Looking for a record in electronic register |
| hosp_admintsk | 13 | SPT:Reading for a record in electronic register |
| hosp_admintsk | 14 | SPT:Retreaving record in electronic register |
| hosp_admintsk | 15 | SPT:filling a new card form electrionic register |
| hosp_admintsk | 16 | SPT:Preparing other monthly summaries (NOT ANC PNC LABOR) |
| hosp_admintsk | 17 | Computer (HC only): |
| hosp_admintsk | 18 | Computer: (HC only): GoTHOMIS data entry |
| hosp_admintsk | 19 | Reading administrative reports |
| hosp_admintsk | 20 | Other work related |
| hosp_admintsk | 21 | Talking to colleague personal |
| hosp_admintsk | 22 | Talking to community health workers on QUADS |
| hosp_admintsk | 23 | Talking to other professionals |
| hosp_admintsk | 24 | Talking to other professionals in person |
| hosp_admintsk | 25 | Filling other forms NOT ANC/Labor/PNC |
|  |  |  |
|  |  |  |
| direct_care_labor | 1 | Pelvic examination |
| direct_care_labor | 2 | Taking vital signs |
| direct_care_labor | 3 | Inserting cannula |
| direct_care_labor | 4 | Setting up drip |
| direct_care_labor | 5 | Abdominal examination |
| direct_care_labor | 6 | Talking to patient |
| direct_care_labor | 7 | Talking to family |
| direct_care_labor | 8 | Talking to patient and family or other accompanying person |
| direct_care_labor | 9 | Taking blood |
| direct_care_labor | 10 | Preparing delivery kit |
| direct_care_labor | 11 | Performing vaginal delivery |
| direct_care_labor | 12 | Preparing vacuum delivery |
| direct_care_labor | 13 | Performing vacuum delivery |
| direct_care_labor | 14 | Preparing caesarean section |
| direct_care_labor | 15 | Performing caesarean section |
| direct_care_labor | 16 | Conducting third stage of labour |
| direct_care_labor | 17 | Cleaning patient |
| direct_care_labor | 18 | Assisting with breast feeding |
| direct_care_labor | 19 | Checking fetal heart rate |
| direct_care_labor | 20 | Inserting urinary catheter |
| direct_care_labor | 21 | Checking urine for protein |
| direct_care_labor | 22 | Checking oxygen saturation |
| direct_care_labor | 23 | Caring for newborn |
| direct_care_labor | 24 | Resuscitating newborn |
| direct_care_labor | 25 | Preparing other direct care interventions (giving injections, inserting catheter, inserting iv line) |
| direct_care_labor | 26 | Giving injection im |
| direct_care_labor | 27 | Inserting iv line |
| direct_care_labor | 28 | Giving injection iv |
| direct_care_labor | 29 | Looking for equipment |
| direct_care_labor | 30 | Stitching |
| direct_care_labor | 31 | Dressing and remove stitches |
|  |  |  |
| direct_care_ANC | 1 | Taking vital signs |
| direct_care_ANC | 2 | Talking to patient |
| direct_care_ANC | 3 | Talking to husband |
| direct_care_ANC | 4 | Talking to patient and husband or other accompanying person |
| direct_care_ANC | 5 | Talking to other accompanying person |
| direct_care_ANC | 6 | HIV/ FP counselling |
| direct_care_ANC | 7 | Taking blood |
| direct_care_ANC | 8 | Doing pelvic examination |
| direct_care_ANC | 9 | Doing abdominal examination |
| direct_care_ANC | 10 | Checking fetal heart rate |
| direct_care_ANC | 11 | Checking urine for protein |
| direct_care_ANC | 12 | Patient education (group) |
| direct_care_ANC | 13 | Measuring weight |
| direct_care_ANC | 14 | Measuring height |
| direct_care_ANC | 15 | Dispensing drugs |
| direct_care_ANC | 16 | Speculum examination |
| direct_care_ANC | 17 | General physical examination |
| direct_care_ANC | 18 | Looking for equipment (e.g. blood pressure cuff) |
| direct_care_ANC | 19 | Providing/inserting fp method |
| direct_care_ANC | 20 | Remving Implant/IUCD |
|  |  |  |
| indirect_care | 1 | Reading: Patient chart/card |
| indirect_care | 2 | Reading: medical guidelines |
| indirect_care | 3 | Writing: Chart notes |
| indirect_care | 4 | Writing: Orders or prescriptions |
| indirect_care | 5 | Writing: Filling partograph |
| indirect_care | 6 | Writing: Issuing ANC card |
| indirect_care | 7 | Writing: Updating information on ANC card |
| indirect_care | 8 | Talking: Discussing patient with colleagues for decision making |
| indirect_care | 9 | Writing: Referral form/letter |
| indirect_care | 10 | Phone: Organizing referral |
| indirect_care | 11 | Cleaning labour room post delivery |
| indirect_care | 12 | Order lab examinations |
| indirect_care | 13 | Review lab results |
| indirect_care | 14 | Writing: documenting delivery |
| indirect_care | 15 | Searching: Lab results |
| indirect_care | 16 | Analysing partograph |
| indirect_care | 17 | Decontamination |
|  |  |  |
| waiting | 1 | Waiting for patient |
| waiting | 2 | Waiting for computer |
| waiting | 3 | Waiting for lab result |
| waiting | 4 | Waiting for colleague |
| waiting | 5 | Waiting for phone call |
| waiting | 6 | Waiting for other work related |
| waiting | 7 | Waiting for Not sure |
|  |  |  |
| walking | 1 | Walking inside labour room |
| walking | 2 | Walking Outside labour room |
| walking | 3 | Walking Inside RCH |
| walking | 4 | Walking Outside RCH |
| walking | 3 | Walking Inside PNC |
| walking | 4 | Walking Outside PNC |
| walking | 5 | Walking - Bringing specimen to lab |
| walking | 6 | Walking -Looking for equipment |
|  |  |  |
| miscelaniuos | 1 | Miscellaneous: Cannot find entry |
| miscelaniuos | 2 | Miscellaneous: Observer personal hygiene |
| miscelaniuos | 3 | Miscellaneous: Work related |
| miscelaniuos | 4 | Phone: not sure |
| miscelaniuos | 5 | Read: Non-medical |
| miscelaniuos | 6 | Talking: Not sure |
| miscelaniuos | 7 | Walking inside or outside other than RCH or labour room |
| miscelaniuos | 8 | Staff shift handover |
|  |  |  |
| gender | 1 | Male |
| gender | 2 | Female |
|  |  |  |
| cadre | 1 | EN |
| cadre | 2 | CO |
| cadre | 3 | AMO |
| cadre | 4 | ANO |
| cadre | 5 | MD |
| cadre | 6 | MA |
| cadre | 7 | ACO |
| cadre | 8 | Other |
|  |  |  |
|  |  |  |
| pnc_tasks | 1 | Taking vital signs |
| pnc_tasks | 2 | Physical examination of patient |
| pnc_tasks | 3 | Assisting with breast feeding |
| pnc_tasks | 4 | Preparing other direct care interventions (giving injections, inserting/removing catheter, inserting iv line, taking blood) |
| pnc_tasks | 5 | Looking for equipment |
| pnc_tasks | 6 | Talking to patient |
| pnc_tasks | 7 | Talking to family |
| pnc_tasks | 8 | Talking to patient and family or other accompanying person |
| pnc_tasks | 9 | Checking urine for protein |
| pnc_tasks | 10 | Newborn: Physical examination |
| pnc_tasks | 11 | Newborn: Cord care |
| pnc_tasks | 12 | Newborn: Vital signs |
| pnc_tasks | 13 | Newborn: Take blood |
| pnc_tasks | 14 | Newborn: Inserting iv line |
| pnc_tasks | 15 | Newborn: Injecting drugs |
| pnc_tasks | 16 | Newborn: Vaccination (BCG, OPV0) |
| pnc_tasks | 17 | Newborn: Weighing |
| pnc_tasks | 18 | Newborn: Assisting with Kangaroo (HC and dispensary only) |
| pnc_tasks | 19 | Dressing and remove stitches |
| pnc_tasks | 20 | councelling on (BF/care fo the new born/FP) |
|  |  | removing catheter/ drip |
