# Supplementary material for: Feasibility, usability and acceptability of a novel digital hybrid-system for reporting of routine maternal health information in Southern Tanzania: A mixed-methods study
Source: PLOS Glob Public Health. 2023 Jan 12;3(1):e0000972. doi: 10.1371/journal.pgph.0000972 (PMC10021923; doi:10.1371/journal.pgph.0000972)
Supplement: S2 Table — (DOCX) [file pgph.0000972.s005.docx]

**S5_Table 3: Overview of themes and categories**

| **Themes** | **Categories** | **Sub-Categories** |
| --- | --- | --- |
| Theme 1: Technology  shaping human interaction | Technical factors influencing data processes | Challenges with SPT system use |
|  |  | Benefits of SPT system design |
|  |  | Challenges with HMIS system |
|  | Technical factors shape health care providers’ practices | Strengthened accountability |
|  | | |
| Theme 2: Human interaction  shaping the use of technology | Health care providers’ capacities shaping use of technology | Challenges with abilities to analyze data |
|  |  | HCPs support implementation of SPT |
|  | Data culture shaping data generation and use | HCPs not empowered to use data |
|  |  | Managers’ views on data |
|  | Organizational factors influencing use of SPT | Contextual challenges affecting SPT use |
|  |  | Challenges associated to lack of training |
|  |  | Support systems already in place |
|  |  | Responsiveness to innovation |
|  |  | Support of facility leadership to SPT |
|  |  | District managers’ support to Institutionalizing SPT |
|  | | |
| Theme 3: Technology and human interaction shaping SPT performance | Improved data collection processes | HCPs supporting each other to implement SPT |
|  |  | Supporting data quality |
|  | Individual and organizational practices influence SPT output | Duplicate data entry is a burden |
|  |  | Potential use of SPT data |
|  | SPT use influences other processes | Improved service performance |
