# Supplementary material for: Feasibility, usability and acceptability of a novel digital hybrid-system for reporting of routine maternal health information in Southern Tanzania: A mixed-methods study
Source: PLOS Glob Public Health. 2023 Jan 12;3(1):e0000972. doi: 10.1371/journal.pgph.0000972 (PMC10021923; doi:10.1371/journal.pgph.0000972)
Supplement: S3 Table — (DOCX) [file pgph.0000972.s006.docx]

**S6_Table 4: Distribution of shifts observed before/after SPT introduction**

| **Potential confounding variables** | | **Number of observed shifts** | | **p-value ^+^** | **Median observed duration in min by shift (Interquartile range)** | |
| --- | --- | --- | --- | --- | --- | --- |
|  |  | **pre- SPT intervention (n= 50)** | **post- SPT intervention (n= 43)** |  | **pre- SPT intervention** | **post- SPT intervention** |
| **Level of care** | Dispensary | 9 | 7 | 0.651 | 144.9 (126.3 – 154.3) | 145.8 (117.0 – 176.7) |
|  | Health centre | 17 | 11 |  | 332.9 (263.9 – 392.3) | 333.7 (289.2 – 390.2) |
|  | District hospital | 24 | 25 |  | 336.0 (301.6 – 364.8) | 337.5 (305.1 – 363.5) |
| **Department** | Antenatal care clinic | 29 | 12 | 0.005* | 309.2 (154.3 – 364.2) | 297.1 (212.2 – 367.2) |
|  | Labour ward | 15 | 16 |  | 332.9 (291.8 – 350.6) | 339.7 (321.5 – 379.5) |
|  | Postnatal care ward | 6 | 11 |  | 325.4 (271.2 – 365.4) | 310.7 (286.9 – 351.1) |
| **Cadre** | Nursing diploma | 25 | 26 | 0.498 | 333.5 (293.9 – 395.2) | 339.7 (310.7 – 381.2) |
|  | Nursing certificate | 20 | 15 |  | 230.9 (143.4 – 327.5) | 255.2 (145.9 – 307.7) |
|  | Other | 5 | 2 |  | 351.2 (346.3 – 356.8) | 317.6 (264.2 – 370.9) |
| **Professional experience** | 1-2 years | 22 | 13 | 0.233 | 269.6 (145.1 – 346.3) | 197.8 (145.9 – 289.2) |
|  | 3-4 years | 26 | 25 |  | 332.9 (277.0 – 364.2) | 339.1 (311.4 – 363.5) |
|  | > 5 years | 2 | 5 |  | 810.8 (438.4 – 1,183.2) | 310.7 (305.1 – 370.9) |
| **Total** | | 50 | 43 |  | 327.5 (239.8 – 362.1) | 317.2 (264.2 – 363.5) |

* Statistical significance, ^+^ Fisher exact test
